# Phase-adaptive automated whole-liver quantification of signal intensity-derived liver indices from gadoxetic acid-enhanced MRI

**DOI:** 10.1186/s41747-026-00813-z

**Published:** 2026-10-12

**Authors:** Felix Barajas Ordonez, Simon Westfechtel, Fadi Matar, Christian Schmidt, Jonathan Lemessa, Patrick Wienholt, Daniel Truhn, Christiane Kuhl, Alexandra Barabasch, Sven Nebelung

**Affiliations:** Department of Diagnostic and Interventional Radiology, University Hospital RWTH Aachen, Aachen, Germany

**Keywords:** Magnetic resonance imaging, Gadoxetic acid, Deep learning, Liver cirrhosis, Image processing, Computer-assisted

## Abstract

**Objectives:**

To develop and validate a phase-adaptive pipeline for automated whole-liver, parenchyma-based quantification of signal intensity-derived liver indices from gadoxetic acid-enhanced MRI without inter-phase co-registration.

**Materials and methods:**

In this retrospective, single-center study (2016–2025), 9,013 liver MRI examinations were screened, and 3,112 gadoxetic acid-enhanced MRI examinations were identified. The first examination per patient defined the clinical cohort (1,174 patients; mean age, 59.6 ± 14 years [range, 4-93 years]; 611 men). A phase-adaptive nnU-Net was trained on 403 and tested on 500 dynamic contrast-enhanced MRI sequences for segmentation of liver parenchyma, intrahepatic non-parenchymal structures, and spleen; paraspinal muscle reference regions were obtained using TotalSegmentator. Segmentation accuracy was assessed against manually corrected reference segmentations generated by a semi-automatic annotation workflow with Dice similarity coefficients. Automated arterial enhancement fraction (AEF), relative liver enhancement (RLE), hepatocellular uptake index (HUI), liver-to-spleen ratio (LSR), and liver-to-muscle ratio (LMR) were compared with manual region-of-interest measurements in a radiological validation cohort (50 patients) using intraclass correlation coefficients and Bland-Altman analysis. Liver indices were compared between patients with cirrhotic and non-cirrhotic livers using the Mann–Whitney *U*-test.

**Results:**

Liver parenchyma segmentation achieved Dice similarity coefficients of 0.998–0.999. Reader-model agreement for the liver indices ranged from 0.936 to 0.977. HUI, LSR, RLE, and LMR were lower in cirrhosis (*p* < 0.001), whereas AEF did not differ (*p* = 0.14).

**Conclusion:**

Phase-adaptive segmentation enables whole-liver quantification of signal intensity-derived liver indices without inter-phase co-registration. The lower hepatobiliary uptake indices in cirrhosis support the plausibility of this approach for scalable quantitative assessment of liver MRI.

**Key Points:**

***Question*** Can gadoxetic acid-enhanced liver MRI be quantified automatically across contrast phases without inter-phase co-registration or manual region-of-interest placement in routine clinical imaging?

***Findings*** Phase-adaptive segmentation enabled automated whole-liver quantification with liver Dice coefficients of 0.998–0.999 and excellent agreement with manual measurements for five liver indices.

***Relevance Statement*** Automated whole-liver quantification from standard gadoxetic acid-enhanced liver MRI provides reproducible, signal-intensity-derived liver indices and reduces dependence on manual region-of-interest measurements in large-scale clinical studies.

**Graphical Abstract:**

**Figure:**
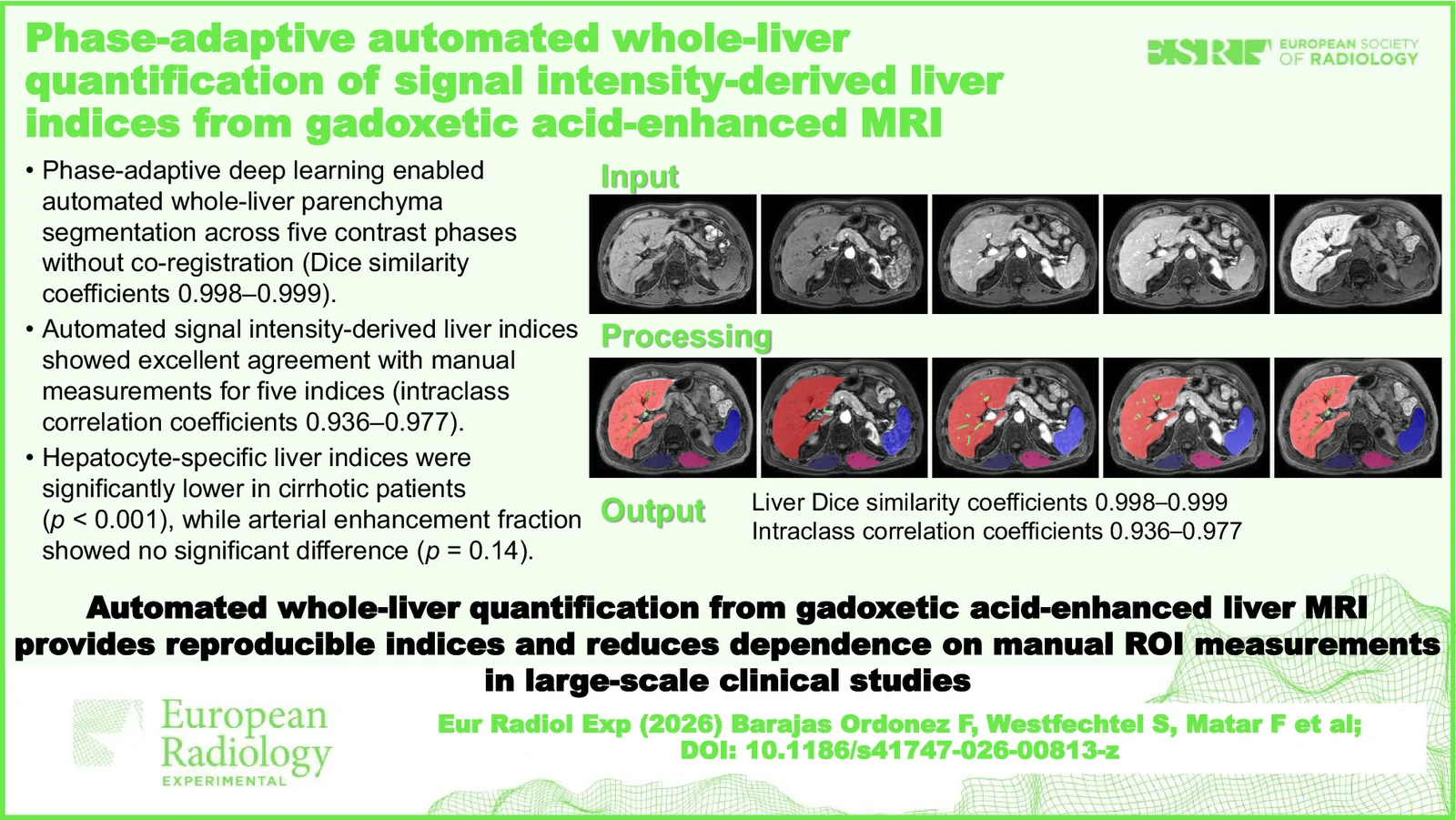

## Introduction

Gadoxetic acid-enhanced liver MRI yields signal intensity (SI)-derived liver indices that reflect hepatocellular uptake and perfusion, including relative liver enhancement (RLE), hepatocellular uptake index (HUI), liver-to-spleen ratio (LSR), and arterial enhancement fraction (AEF) [1, 2]. These liver indices have been associated with liver dysfunction and fibrosis [3–6]. However, their use in routine imaging remains limited because they usually depend on manual region-of-interest placement and phase-specific post-processing.

Deep learning has improved automated liver segmentation, but most published approaches address a single contrast phase or require inter-phase co-registration for multi-phase analysis [7–9]. In gadoxetic acid-enhanced dynamic imaging, respiratory motion, variable breath-hold positions, and non-rigid liver deformation make reliable alignment across phases difficult and can propagate error into quantitative measurements [10]. A robust method for automated multi-phase quantification without co-registration remains lacking [11].

This retrospective study aimed to develop and validate a phase-adaptive segmentation pipeline for automated whole-liver, parenchyma-based quantification of SI-derived liver indices from standard gadoxetic acid-enhanced MRI without inter-phase co-registration. By *phase-adaptive*, we refer to a model that works robustly across all liver dynamic contrast-enhanced (DCE) phases. We hypothesized that (i) such an approach would achieve high segmentation accuracy for liver parenchyma and intrahepatic non-parenchymal structures across phases, (ii) automated SI-based liver indices would show high agreement with manual region-of-interest-based measurements, and (iii) hepatobiliary phase (HBP)-derived liver indices would be lower in cirrhotic than in non-cirrhotic patients.

## Materials and methods

### Study design and patient selection

This retrospective single-center diagnostic accuracy study was approved by the institutional review board (EK 24-148, approved 25 April 2024), and the requirement for written informed consent was waived. Consecutive gadoxetic acid-enhanced liver MRI examinations were retrieved from our institutional archive. The study comprised three parts: (i) technical validation of a phase-adaptive automated segmentation pipeline against reference segmentations, (ii) within-patient comparison of automated *versus* manual region of interest (ROI)-based liver indices, and (iii) application of the final pipeline to the first eligible examination per patient in the clinical cohort.

The study was conducted at a tertiary academic hepatobiliary referral center. All liver DCE-MRI examinations performed between January 2016 and May 2025 were manually screened using the institutional Picture Archiving and Communication System (Version 12.2.8.410; Philips Vue Healthcare). Examinations were considered eligible if gadoxetic acid had been administered. Examinations without contrast administration, without a complete set of DCE sequences (from the arterial to the HBP), or performed with other contrast agents were excluded. The presence of hepatic lesions, regardless of lesion size or infiltrative growth pattern, did not constitute a cohort exclusion criterion.

**Fig. 1:**
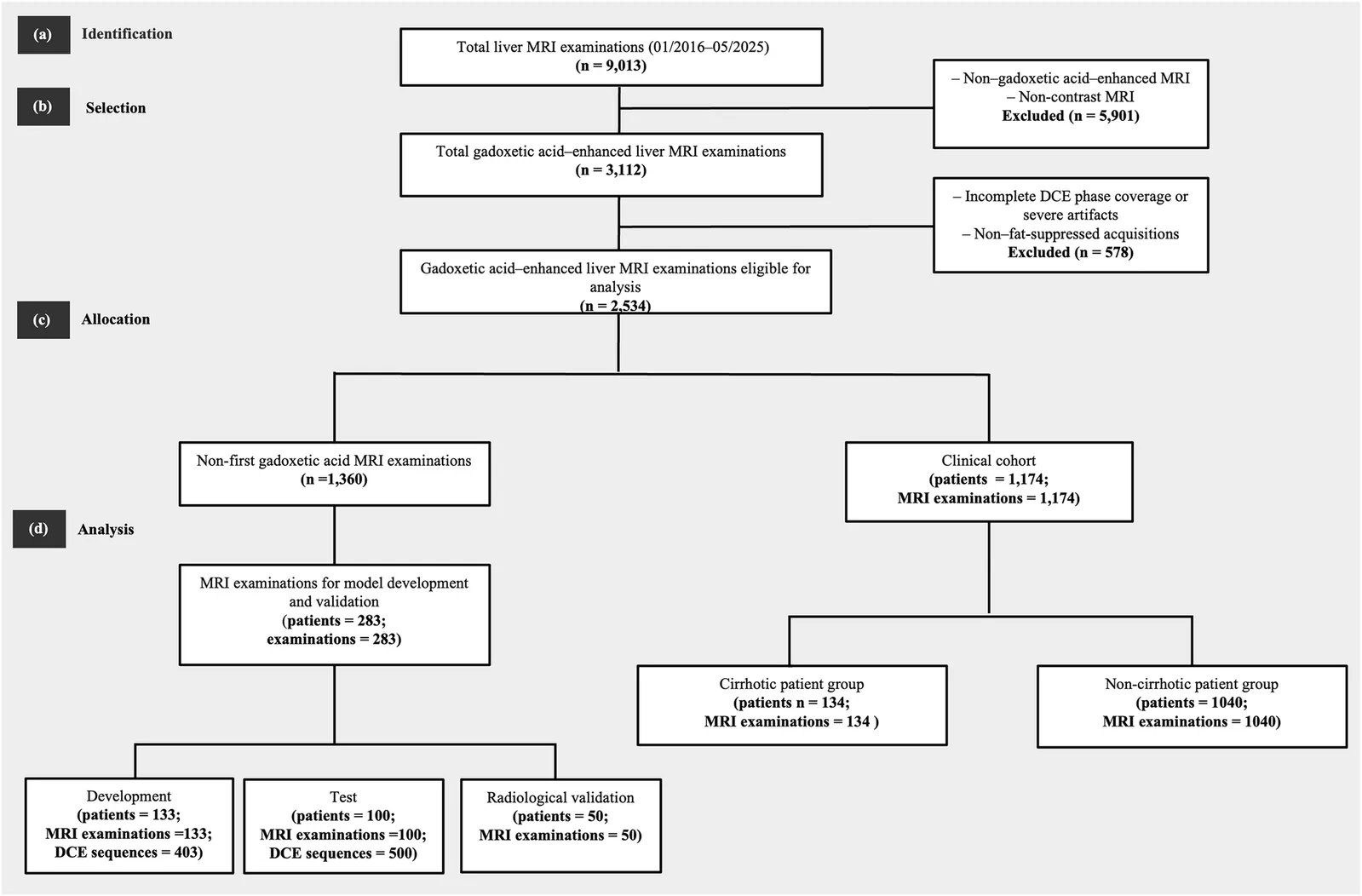
Study flowchart and cohort allocation. Of 9,013 liver MRI examinations acquired between January 2016 and May 2025, 3,112 gadoxetic acid-enhanced MRI examinations were identified. After exclusion of examinations with incomplete DCE phase coverage, severe artifacts, or non-fat-suppressed acquisitions, 2,534 examinations remained. Of these, 1,174 first MRI examinations per patient constituted the clinical cohort (1,174 patients; 1,174 MRI examinations), including a cirrhotic subgroup (134 patients; 134 MRI examinations) and a non-cirrhotic subgroup (1,040 patients; 1,040 MRI examinations). Additional non-first MRI examinations were used for model development, test cohort, and radiological validation (development cohort: 133 patients, 133 MRI examinations, and 403 annotated DCE sequences; test cohort: 100 patients, 100 MRI examinations, and 500 annotated DCE sequences; radiological validation cohort: 50 patients and 50 MRI examinations); the remaining examinations were not further included in the analysis

Among all eligible gadoxetic acid-enhanced examinations, the first examination per patient was identified (hereafter referred to as the “clinical cohort”). Additional gadoxetic acid-enhanced examinations from the same patients were used for model development (development and test cohort) and for radiological validation (radiological validation cohort). Assignment to the development, test, and radiological validation cohorts was performed randomly at the patient level by a computational scientist (SW), blinded to clinical data. No patient contributed to more than one of the development, test, and radiological validation cohorts. Non-first examinations not assigned to any of these cohorts were excluded from further analysis. Within the clinical cohort, patients were categorized as cirrhotic or non-cirrhotic based on information extracted from radiology reports and clinical records. Cirrhosis was defined by explicit documentation of cirrhosis or by radiology reports describing imaging findings compatible with cirrhotic liver morphology, including nodular liver contour or segmental or lobar volume redistribution with hypertrophy or atrophy. Signs of portal hypertension, such as splenomegaly, ascites, or portosystemic collaterals, were considered supportive findings but were not used as stand-alone criteria. Patients without documented cirrhosis or reported cirrhotic liver morphology were categorized as non-cirrhotic. MRI examinations with severe artifacts, non-fat-suppressed DCE sequences, or incomplete DCE acquisitions were excluded (Fig. 1).

### Clinical data sources and radiological report assessment

Clinical variables were extracted from the corresponding radiological reports and the institutional information system by a radiology resident with 5 years of experience (FBO), including age, sex, MRI indication, cirrhosis status, and splenectomy status.

### MRI protocol

**Fig. 2:**
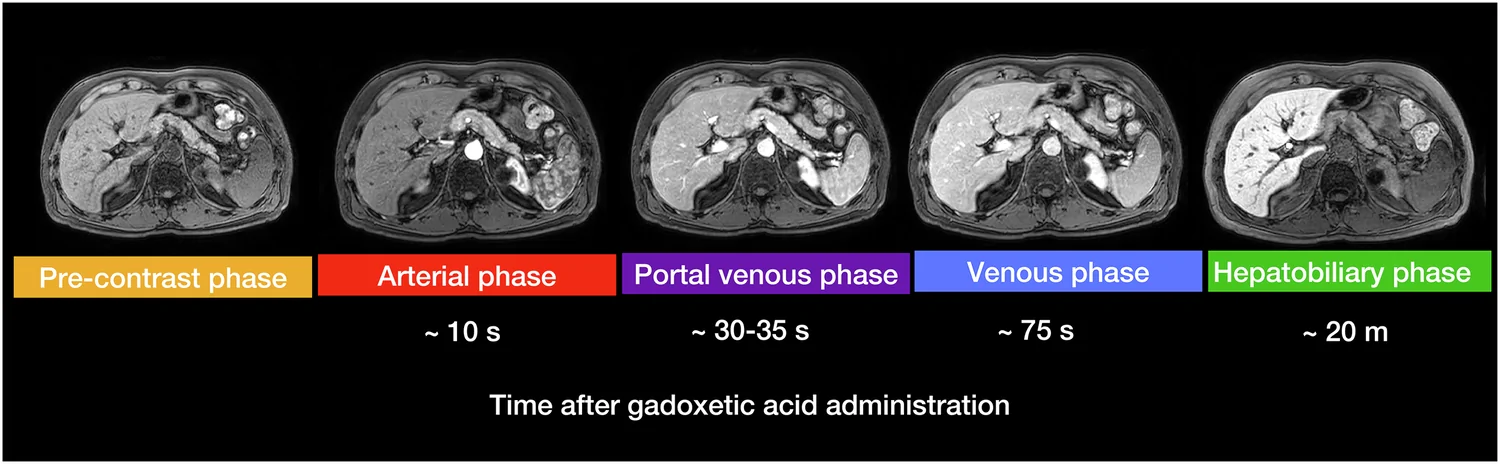
Representative DCE MRI phases after gadoxetic acid administration. Axial T1-weighted fat-suppressed images illustrate the pre-contrast, arterial, portal venous, venous, and hepatobiliary phases used for automated analysis. Images are shown in their temporal acquisition order after gadoxetic acid administration

Our MRI protocol has been described previously [12–14]. All MRI examinations were performed on 1.5-T systems (Ambition or Ingenia; Philips Healthcare) using a dedicated 16-channel phased-array torso receiver coil (SENSE XL Torso Coil, Philips Healthcare) with standardized acquisition protocols across systems (Supplementary Table S1). The protocol included an axial T1-weighted gradient echo sequence (in-phase and opposed-phase), axial diffusion-weighted imaging with b-values of 0, 400, and 800 s/mm² and corresponding apparent diffusion coefficient maps, an axial T2-weighted turbo spin-echo sequence with and without fat saturation, and a coronal T2-weighted turbo spin-echo sequence. Gadoxetic acid (Primovist, Bayer Healthcare) was administered intravenously at a dose of 0.025 mmol/kg body weight (0.1 mL/kg at a concentration of 0.25 mmol/mL) using a power injector at a flow rate of 2 mL/s, followed by a saline flush. DCE sequences were acquired in the axial plane using a T1-weighted gradient echo sequence, including a pre-contrast (PC) phase immediately before contrast administration, followed by the arterial phase (AP), portal venous phase (PVP), and venous phase (VP) obtained at approximately 10, 30–35, and 75 s post-injection, respectively. An additional HBP was obtained 20 min after injection (Fig. 2). No transitional-phase acquisitions were obtained.

### Phase-adaptive segmentation and quantification framework

A modular, phase-adaptive automated pipeline was implemented, integrating liver segmentation with exclusion of intrahepatic non-parenchymal structures, signal normalization using spleen and paraspinal muscle references, and parenchyma-based quantification of SI-derived liver indices. A 3D nnU-Net (nnU-Net 2.5.1, PyTorch version 2.2.1) served as the segmentation backbone of the pipeline. Deviations from the default nnU-Net implementation comprised additional, MRI-specific data augmentation (*e.g.*, simulated motion artifacts) [15]. The network was initialized with weights pre-trained on the TotalSegmentator MRI dataset [16], comprising 616 MRI studies across various anatomical regions. Since TotalSegmentator does not support all relevant intrahepatic structures, and exact delineation between anatomical structures was necessary for accurate extraction of parenchymal SI, the model was subsequently fine-tuned on internal DCE-MRI studies, tailoring it towards accurate segmentation of liver parenchyma, intrahepatic non-parenchymal structures, and the spleen. Pre-processing of all data was automatically handled by nnU-Net and included cropping images to non-zero regions, resampling to a common voxel spacing, and *z*-score normalization.

### Model development, staged fine-tuning, and test cohort evaluation

Model development followed a staged phase-expansion strategy rather than repeated ad-hoc retraining. The externally pre-trained weights described above were first adapted to the PVP because this phase provided the most consistent contrast between liver parenchyma and intrahepatic non-parenchymal structures. To this end, 40 PVP reference sequences from 40 patients were used to train an initial binary model separating liver parenchyma from intrahepatic non-parenchymal structures. Next, this initialization was fine-tuned on 68 paired PVP and VP sequences from 34 patients to improve robustness to late dynamic contrast variation. Thereafter, the training set was expanded to 295 additional sequences from 59 patients spanning all five dynamic phases (PC, AP, PVP, VP, and HBP), yielding the final phase-adaptive model. In total, model development used 403 annotated sequences from 133 patients, with no overlap of patients between training stages.

Reference segmentations were generated using a semi-automatic annotation workflow. Initial liver and spleen masks were produced with TotalSegmentator, a model independent of the final phase-adaptive model and not trained on the test cohort. These initial masks were used only to accelerate annotation and did not serve as the reference standard. All masks were subsequently reviewed and manually corrected, slice by slice in 3D Slicer (version 5.10) by a pre-graduate medical student (FM), including correction of liver and spleen boundaries as well as manual annotation of intrahepatic non-parenchymal structures, according to a prespecified annotation protocol. The corrected masks were then reviewed by FBO, and only the fully corrected segmentations were used as reference segmentations for training and test-set evaluation. Test-set reference segmentations were generated before final model evaluation and were not used for model fitting, hyperparameter tuning, or final model selection.

For the final model, target labels comprised liver parenchyma, intrahepatic non-parenchymal structures, and the spleen. The non-parenchymal class included intrahepatic vessels, bile ducts, focal liver lesions, and non-functional post-treatment changes, including resection cavities and ablation zones, which were excluded from the parenchymal mask. Structures smaller than 3 mm were not labeled separately because reliable delineation was limited by the effective spatial resolution and partial-volume effects.

Dataset partitioning was performed at the patient level prior to model development to prevent data leakage, and no patient contributed to more than one of the development, test, and radiological validation cohorts. The test cohort comprised 500 reference sequences from 100 patients with complete phase coverage (PC, AP, PVP, VP, and HBP). Test-set reference segmentations were generated using the same protocol as in the development set and were not used for model fitting, hyperparameter tuning, or final model selection.

All training stages used the same base nnU-Net configuration (initial learning rate, 0.01; optimizer, stochastic gradient descent; learning-rate schedule, polynomial decay; loss function, soft-Dice plus cross-entropy; 1,000 epochs). On-the-fly augmentation included random rotation, mirroring, cropping, blurring, and simulated motion artifacts. For training and validation, we used five-fold cross-validation. The final deployed model used for test-set evaluation and cohort-wide application was an ensemble of all five validation folds. Training and inference were performed with nnU-Net version 2.5.1 and PyTorch version 2.2.1 on a dedicated high-performance computing node with an H100 GPU; mean inference time was 36 ± 4 s per sequence and 180 ± 20 s per complete examination.

### Automated whole-liver quantification of SI-derived liver indices

Parenchyma-based SI values were extracted from the parenchyma-only liver mask for each DCE phase without inter-phase co-registration. The axial slice with the largest cross-sectional area of the spleen was identified, and the bilateral paraspinal muscles at that level were segmented using TotalSegmentator [16]. In patients without a spleen, the axial slice containing liver parenchyma with the largest cross-sectional area of paraspinal muscle segmentations was automatically selected as an alternative reference level. Muscle SI was measured using circular ROI centered on the geometric centroids of the segmented paraspinal muscles. The radius of each ROI was set to 25% of the radius of a fitted circle enclosing the respective muscle segmentation, and the resulting mean muscle SI was used for normalization of liver parenchymal SI. Based on these normalized values, SI-derived liver indices were computed from predicted segmentation masks, including RLE (HBP, normalized), AEF, HUI, LSR, and liver-to-muscle ratio (LMR). The segmentation output also enabled automated volumetric quantification of the liver and spleen, which was incorporated into index calculation, particularly for HUI. The SI-derived liver indices used in the present study are defined in Table 1.

**Table 1:** Overview of SI-derived liver indices for automated whole-liver quantification

Index	Phase(s) used	What the index reflects	Formula used in this study	Main strength	Main limitation	Key reference(s)
AEF	AP, PC, PVP	Relative contribution of arterial enhancement compared with portal venous enhancement; perfusion-weighted marker	$\left(\frac{{SI}_{{liver}{AP}}-{SI}_{{liver}{PC}}}{{SI}_{{liver}{PVP}}-{SI}_{{liver}{PC}}}\right)\times 100 \%$	Captures early dynamic enhancement behavior beyond hepatobiliary uptake	Sensitive to timing of AP and PVP acquisition, motion, and injection variability; less directly linked to hepatocyte uptake	[6]
RLE (RLE, HBP, normalized)	PC, HBP	Relative HBP signal increase of the liver after gadoxetic acid administration	$\frac{\left[\left(\frac{{SI}_{{liver}{HBP}}}{{SI}_{{muscle}{HBP}}}\right)-\left(\frac{{SI}_{{liver}{PC}}}{{SI}_{{muscle}{PC}}}\right)\right]}{\left(\frac{{SI}_{{liver}{PC}}}{{SI}_{{muscle}{PC}}}\right)}$	Intuitive and widely used hepatobiliary uptake surrogate	Introduces additional normalization steps (PC and muscle-based normalization), which may increase methodological complexity and dependence on less standardized muscle reference tissue.	[1]
HUI	HBP	Composite uptake marker combining liver signal and liver volume, usually spleen-normalized	${liver}{volume}({mL})\times \left[\left(\frac{{SI}_{{liver}{HBP}}}{{SI}_{{spleen}{HBP}}}\right)-1\right]$	Integrates uptake and liver volume; among the most function-oriented SI-derived indices	More complex than simple ratios; depends on accurate liver volumetry and splenic normalization; potentially limited in splenectomy or altered splenic signal	[1, 3]
LSR	HBP	Relative liver SI normalized to spleen signal	$\frac{{{SI}}_{{liver\; HBP}}}{{{SI}}_{{spleen\; HBP}}}$	Simple, established, and easy to interpret	Requires spleen as reference; potentially limited in splenectomy or altered splenic signal	[1, 5, 17]
LMR	HBP	Relative liver SI normalized to paraspinal muscle signal	$\frac{{SI}_{{liver}{HBP}}}{{{SI}}_{{muscle}{HBP}}}$	Provides an alternative internal reference when spleen-based normalization is unavailable or unreliable	Muscle signal may be less standardized and may be affected by sarcopenia, myopathy, or fatty infiltration	[1, 4, 5]

*AP* Arterial phase, *HBP* Hepatobiliary phase, *PC* Pre-contrast phase, *PVP* Portal venous phase, *SI* Signal intensity

Following model development, internal testing, and radiological validation, the fully trained pipeline was applied to all examinations of the clinical cohort for quantitative imaging analysis. Patients with prior splenectomy were excluded from analyses requiring spleen-based normalization.

### Manual quantitative analysis

**Fig. 3:**
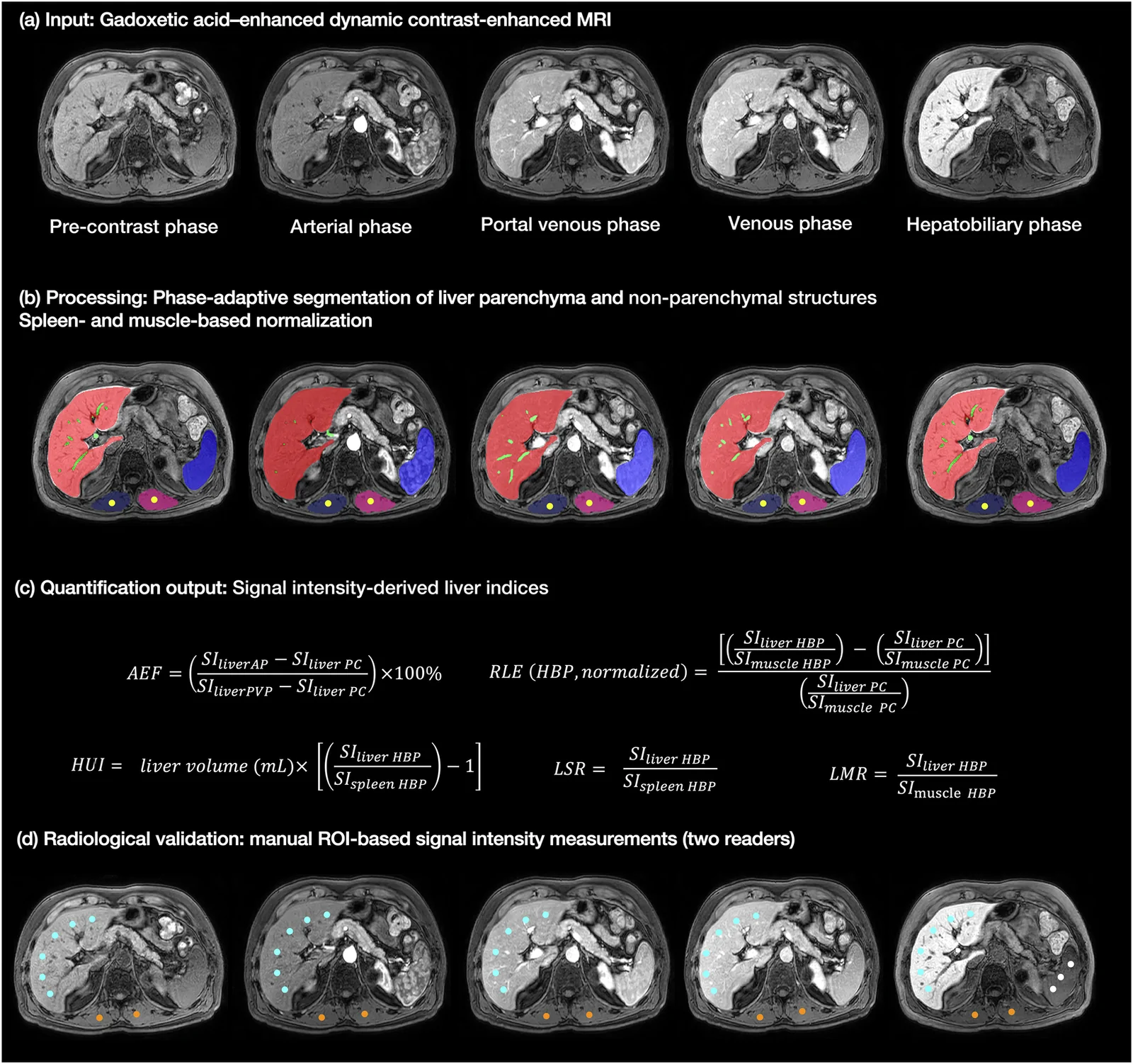
Automated segmentation and quantification workflow with radiological validation. **a** Pre-contrast, arterial, portal venous, venous, and HBP T1-weighted fat-suppressed images served as multi-phase input. **b** The phase-adaptive pipeline segmented liver parenchyma, intrahepatic non-parenchymal structures, and spleen across phases; paraspinal muscle reference regions were obtained separately using TotalSegmentator. Liver parenchyma is shown in red, excluded intrahepatic non-parenchymal structures in green, spleen in blue, paraspinal muscle in purple, and muscle reference regions in yellow. **c** Signal intensities and organ volumes derived from the segmentation masks were used to calculate AEF, RLE, HUI, LSR, and LMR. **d** For radiological validation, two readers performed manual region-of-interest-based SI measurements in corresponding liver, muscle, and spleen regions. PC, Pre-contrast phase; AP, Arterial phase; PVP, Portal venous phase; VP, Venous phase; HBP, Hepatobiliary phase; AEF, Arterial enhancement fraction; RLE, Relative liver enhancement; HUI, Hepatocellular uptake index; LSR, Liver-to-spleen ratio; LMR, Liver-to-muscle ratio

For radiological validation, all manual quantitative measurements were performed in the radiological validation cohort (*n* = 50 patients) using the local PACS by two radiology residents, FBO (5 years of experience) and CS (4 years of experience). SI of the left and right liver lobes was measured using three separate circular ROIs placed in each lobe, avoiding non-parenchymal structures such as large vessels, bile ducts, or tumor masses. ROIs were defined on the same axial slice across all contrast phases [17]. The reference slice was chosen at the level where the spleen showed its largest cross-sectional area. Each circular ROI had an area of 20 mm². On the selected axial slice, the SI of the paraspinal muscles was measured bilaterally (one ROI for each side) [1] and the SI of the spleen was measured in the HBP [18] (three ROIs). Subsequently, the mean SI of the liver, spleen, and paraspinal musculature was calculated, and SI-derived liver indices were computed using formulas analogous to those implemented in the post-processing algorithm (Fig. 3).

### Statistical analysis

Continuous variables are reported as mean ± standard deviation (SD) or median [interquartile range (IQR)], as appropriate, and categorical variables as counts with percentages. Segmentation performance in the test cohort was evaluated using the Dice similarity coefficient (DSC) and average surface distance. All analyses were performed on a complete-case basis. For liver indices requiring spleen-based normalization (HUI and LSR), patients with prior splenectomy were excluded from the respective analyses. Baseline characteristics of the development and test cohorts were compared using the Student’s *t*-test or Fisher’s exact test, as appropriate. Interreader agreement and reader-model agreement for SI-derived liver indices in the radiological validation cohort were assessed using intraclass correlation coefficients (ICC) with 95% confidence intervals, based on a two-way random-effects model for single measurements with absolute agreement. ICC values were interpreted as poor (< 0.50), moderate (0.50–0.75), good (0.75–0.90), and excellent (> 0.90) [19]. Agreement was further assessed using Bland-Altman analysis, including estimation of mean differences (bias) and 95% limits of agreement. In the clinical cohort, automated liver indices were compared between cirrhotic and non-cirrhotic patients using the Mann–Whitney *U*-test. All statistical analyses were performed using R (version 4.3.2; R Foundation for Statistical Computing). Two-sided *p*-values < 0.05 were considered statistically significant.

## Results

### Study population

Of 9,013 liver MRI examinations screened, 3,112 gadoxetic acid-enhanced MRI examinations were identified. After exclusion of examinations with incomplete phase coverage, severe artifacts, or non-fat-suppressed acquisitions, 2,534 examinations remained. Of these, 1,174 first examinations per patient constituted the clinical cohort (1,174 patients; mean age, 59.6 ± 14 years [range, 4–93 years], 611 men), including a cirrhotic patient group (134 patients; 134 MRI examinations) and a non-cirrhotic patient group (1,040 patients; 1,040 MRI examinations). Full baseline characteristics of the clinical cohort are provided in Table 2.

**Table 2:** Baseline characteristics of the clinical cohort (*n* = 1,174)

Characteristic	Data are *n* (%) unless otherwise indicated
Age (years) mean ± SD (range)	59.6 ± 14 (4–93)
Male sex	611 (52.0%)
MRI indication	
Follow-up	664 (56.6%)
Diagnostic work-up	500 (42.6%)
Other	10 (0.9%)
Underlying liver disease and portal hypertension	
Hepatic steatosis	94 (8.0%)
Cirrhosis	134 (11.4%)
Ascites	121 (10.3%)
Splenomegaly	123 (10.5%)
Portal hypertension	63 (5.4%)
Surgical and treatment history	
Previous liver surgery	626 (53.3%)
Hemihepatectomy	78 (6.6%)
Liver transplantation	28 (2.4%)
Biliodigestive anastomosis	38 (3.2%)
Cholecystectomy	130 (11.1%)
Splenectomy	13 (1.1%)
Lesion characteristics and burden	
Hemangioma	76 (6.5%)
FNH	80 (6.8%)
Adenoma	25 (2.1%)
Cystic lesion	203 (17.3%)
Primary malignant lesion	228 (19.4%)
HCC	108 (9.2%)
iCCA	105 (8.9%)
Other	15 (1.3%)
Secondary malignant lesions	516 (44.0%)
Colorectal	323 (27.5%)
Pancreatic	45 (3.8%)
Breast	26 (2.2%)
Other	122 (10.4%)
Lesion burden	
0	113 (9.6%)
1	211 (18.0%)
2	236 (20.1%)
3	75 (6.4%)
Multiple	539 (45.9%)

Data are numbers of patients, with percentages in parentheses, unless otherwise indicated.

Categories may not sum to 100% because multiple findings per patient were possible

*HCC* Hepatocellular carcinoma, *iCCA* Intrahepatic cholangiocarcinoma, *FNH* Focal nodular hyperplasia, *SD* Standard deviation

Additional non-first examinations, randomly allocated at the patient level, were used for model development, test cohort evaluation, and radiological validation. The development and test cohorts comprised 233 patients with 233 MRI examinations, yielding 903 annotated DCE sequences (mean age 60.3 ± 13.2 years [range, 19-87 years], 133 men). No significant differences in baseline characteristics were observed between cohorts (Supplementary Table S2). The radiological validation cohort comprised 50 additional patients with 50 MRI examinations.

### Segmentation performance

**Fig. 4:**
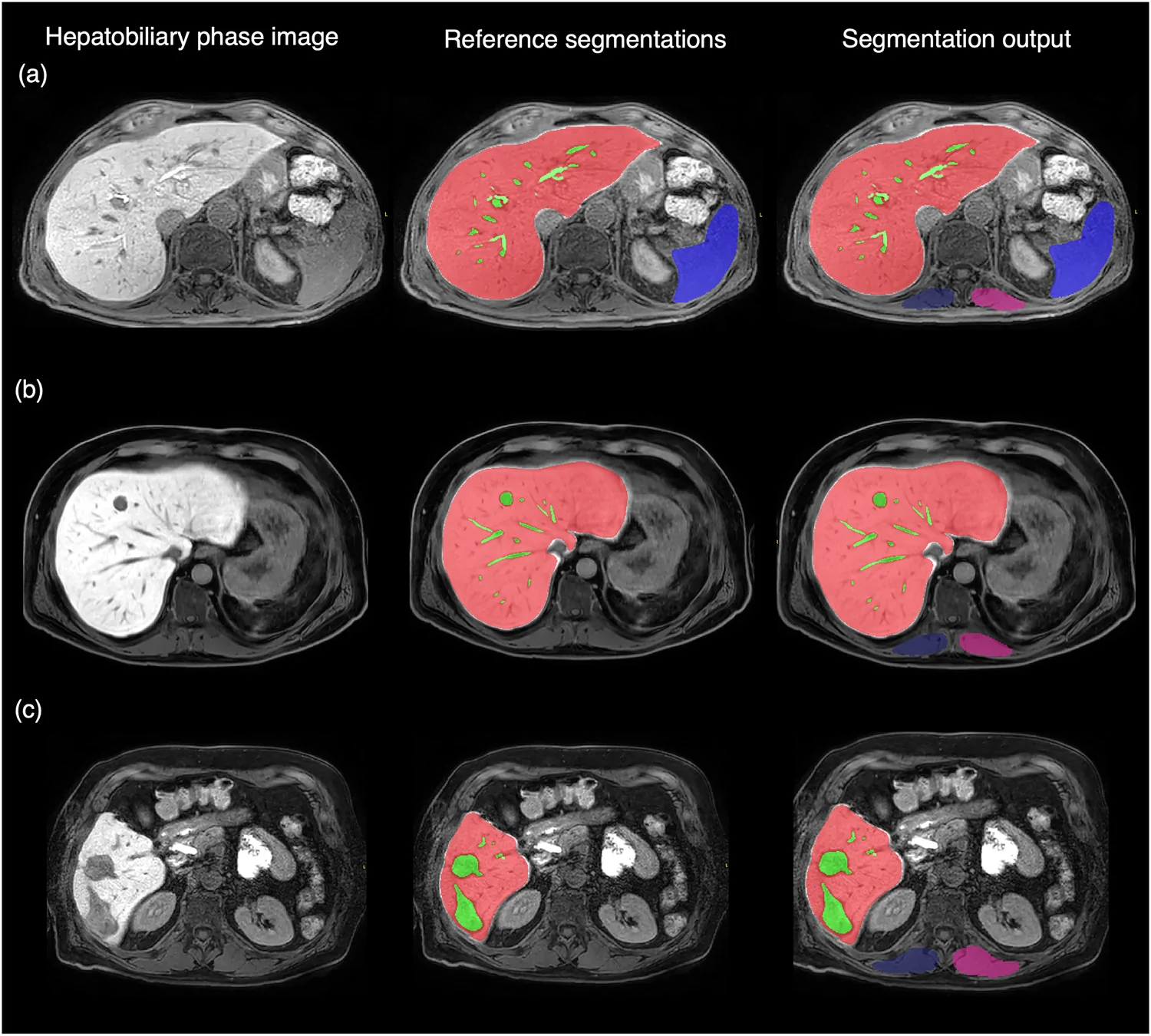
Representative segmentation results in the test cohort. Axial hepatobiliary-phase T1-weighted fat-suppressed MRI images are shown with reference segmentations and automated segmentation outputs. **a** Liver without focal lesions. **b** Single colorectal liver metastasis excluded from the parenchymal mask. **c** Postoperative liver anatomy after left hemihepatectomy with residual tumor burden and reduced parenchymal volume. Liver parenchyma is shown in red, excluded intrahepatic non-parenchymal structures in green, and the spleen in blue. **b**, **c** No spleen is shown due to prior splenectomy. Paraspinal muscle segmentations are shown in the model output only and were used for signal normalization, but were not part of the reference segmentations

Liver parenchyma segmentation accuracy was high across all five contrast phases (Table 3), with median DSC ranging from 0.998 (HBP) to 0.999 (PC, AP, PVP, VP) and median average surface distances below 0.04 mm. Non-parenchymal liver structures showed lower median DSC values (0.873–0.945); the highest DSC was observed in the PVP, where portal venous contrast maximized vessel-parenchyma differentiation. The lower DSC for non-parenchymal liver structures is consistent with their smaller size and lower boundary conspicuity compared to the large liver parenchyma. Spleen segmentation yielded near-perfect overlap across all phases (median DSC of 1.000) (Fig. 4 and Supplementary Fig. S1). Segmentation performance for liver parenchyma and spleen was better across all phases for the phase-adaptive model than for TotalSegmentator MRI, with higher DSC and lower average surface distances (Supplementary Table S3 and Supplementary Fig. S2).

**Table 3:** Phase-specific segmentation performance in the test cohort based on 500 annotated DCE MRI sequences

	DSC	Average surface distance [mm]
Pre-contrast phase		
Liver parenchyma	0.999 [0.998–0.999]	0.020 [0.007–0.081]
Non-parenchymal liver structures	0.924 [0.860–0.956]	0.387 [0.174–0.926]
Spleen^*^	1.000 [0.988–1.000]	0.000 [0.000–0.175]
AP		
Liver parenchyma	0.999 [0.998–0.999]	0.032 [0.009–0.091]
Non-parenchymal liver structures	0.911 [0.835–0.953]	0.458 [0.210–0.947]
Spleen^*^	1.000 [0.989–1.000]	0.000 [0.000–0.153]
PVP		
Liver parenchyma	0.999 [0.998–0.999]	0.021 [0.005–0.048]
Non-parenchymal liver structures	0.945 [0.895–0.973]	0.270 [0.114–0.564]
Spleen^*^	1.000 [0.998–1.000]	0.000 [0.000–0.022]
VP		
Liver parenchyma	0.999 [0.998–0.999]	0.026 [0.011–0.054]
Non-parenchymal liver structures	0.873 [0.800–0.923]	0.553 [0.320–0.903]
Spleen^*^	1.000 [0.993–1.000]	0.000 [0.000–0.152]
HBP		
Liver parenchyma	0.998 [0.997–0.999]	0.035 [0.010–0.086]
Non-parenchymal liver structures	0.924 [0.857–0.958]	0.332 [0.153–0.727]
Spleen^*^	1.000 [0.924–1.000]	0.000 [0.000–0.109]

Data are median [interquartile range]

The test cohort comprised 100 patients with complete phase coverage and 500 annotated DCE MRI sequences. DSC values range from 0 to 1, with higher values indicating better overlap. Average surface distance is reported in millimeters, with lower values indicating better boundary agreement

^*^ Spleen segmentation metrics were calculated in 96 patients (480 annotated DCE MRI sequences) because four patients had prior splenectomy.

### Interreader and reader-model agreement for SI-derived liver indices

Interobserver agreement between the two radiologists was excellent for all five SI-derived liver indices (ICC 0.960–0.989). Reader-model agreement was similar (ICC 0.936–0.977), with the highest agreement for HUI and the lowest for LMR (Table 4).

**Table 4:** Interreader and reader-model agreement for automated liver indices in the radiological validation cohort (*n* = 50)

	Mean ± standard deviation			ICC [95% confidence interval]		
Index	Reader 1	Reader 2	Model	Reader 1 *versus* Reader 2	Reader 1 *versus* Model	Reader 2 *versus* Model
AEF	39.3 ± 23.5	39.7 ± 23.1	40.4 ± 21.9	0.975 [0.960, 0.990]	0.958 [0.930, 0.980]	0.953 [0.920, 0.970]
RLE (HBP, normalized)	0.86 ± 0.25	0.86 ± 0.25	0.87 ± 0.24	0.960 [0.930, 0.980]	0.971 [0.950, 0.980]	0.949 [0.910, 0.970]
HUI	2014 ± 713	1968 ± 731	1910 ± 683	0.989 [0.980, 0.990]	0.971 [0.880, 0.990]	0.977 [0.950, 0.990]
LSR	2.38 ± 0.44	2.34 ± 0.44	2.29 ± 0.39	0.987 [0.960, 0.990]	0.959 [0.800, 0.990]	0.968 [0.930, 0.980]
LMR	3.05 ± 0.50	3.06 ± 0.51	2.93 ± 0.42	0.984 [0.970, 0.990]	0.945 [0.690, 0.980]	0.936 [0.710, 0.980]

Data are mean ± standard deviation

ICCs were computed using a two-way random-effects model (single measures, absolute agreement) with 95% confidence intervals. The radiological validation cohort comprised 50 patients. Indices were computed per patient by aggregating phase-specific SI values from manually placed regions of interest (Reader 1 and Reader 2) and from the parenchyma-only liver mask (model output)

*HBP* Hepatobiliary phase

Bland-Altman analysis showed small mean differences for RLE (HBP, normalized), LSR, and LMR, with biases close to zero (|bias| ≤ 0.11) and narrow limits of agreement. In contrast, HUI and AEF showed larger mean differences and wider limits of agreement, particularly in reader-model comparisons (Supplementary Table S4 and Supplementary Fig. S3).

### SI-derived liver indices in cirrhotic *versus* non-cirrhotic patients

Patients with cirrhosis showed markedly lower hepatocyte-specific indices compared with patients without cirrhosis, including HUI, LSR, RLE (HBP, normalized), and LMR (all *p* < 0.001). AEF did not differ significantly between groups (*p* = 0.14) (Table 5).

**Table 5:** Comparison of SI-derived liver indices between cirrhotic and non-cirrhotic patients (*n* = 1,174)

Index	Non-cirrhotic patients (*n* = 1,040)	Cirrhotic patients (*n* = 134)	*p*-value
AEF	32.45 [20.64–50.98]	40.57 [16.76–60.02]	0.14
RLE (HBP, normalized)	0.74 [0.49–0.94]	0.44 [0.16–0.60]	< 0.001
HUI^*^	1,535 [1,107–2,049]	682 [242–1,183]	< 0.001
LSR^*^	2.05 [1.77–2.36]	1.50 [1.15–1.80]	< 0.001
LMR	2.65 [2.35–2.98]	2.01 [1.72–2.29]	< 0.001

*HBP* Hepatobiliary phase

^*^ HUI and LSR require spleen-based normalization; 13 non-cirrhotic patients with prior splenectomy were excluded from these analyses (non-cirrhotic *n* = 1,027; cirrhotic *n* = 134; total *n* = 1,161). *p*-values were calculated using the Mann–Whitney *U*-test

## Discussion

This study developed and validated a deep learning-based pipeline for automated segmentation of liver parenchyma, intrahepatic non-parenchymal structures, and the spleen in gadoxetic acid-enhanced MRI. This approach enabled automated whole-liver, parenchyma-based quantification of five SI-derived liver indices without inter-phase co-registration. Automated quantification of these liver indices showed excellent agreement with manual reader-based measurements (ICCs, 0.936–0.977) and was feasible in a large clinical cohort. In addition, HBP-derived liver indices (HUI, LSR, RLE [HBP, normalized], LMR) showed significantly lower values in the cirrhotic than in non-cirrhotic patients, consistent with reduced hepatocellular uptake of gadoxetic acid in chronic liver disease [3]. AEF did not differ between groups, consistent with its dependence on early dynamic enhancement and acquisition-related variability rather than hepatocyte-specific uptake [6].

Prior automated approaches were confined to single contrast phases [7, 8] or required co-registration for multi-phase analysis: Raab et al [9] used nnU-Net with co-registration across DCE phases, and the closest conceptual comparator, Zbinden et al [20], recently introduced a sequence-adaptive pipeline co-registering T1 Dixon labels to other parametric sequences. In contrast, the present approach does not rely on inter-phase alignment. However, it follows the clinical principle of phase-specific assessment, analogous to manual ROI placement by radiologists, who evaluate SI within parenchyma while avoiding intrahepatic non-parenchymal structures. Our staged single-model approach achieves comparable accuracy (median DSC, 0.998–0.999) without alignment, avoiding motion-induced spatial errors in breath-hold-dependent acquisitions. Previous studies mostly focused on single indices derived from individual contrast phases (*e.g.*, HBP LSR [8]), whereas the present approach enables automated whole-liver quantification of multiple indices across DCE phases. The LMR, previously described in manual ROI-based studies [21], was computed automatically using ROI measurements derived from automated muscle segmentation. SI-derived liver indices have been associated with liver dysfunction and fibrosis [3–6], and consistent with this literature, HBP-derived indices in the present study significantly differentiated cirrhotic from non-cirrhotic patients. Notably, this differentiation was demonstrated across the full clinical cohort of 1,174 patients, a scale that would be impractical with manual ROI placement, despite the excellent reader-model agreement already reported above. The primary improvement over conventional manual assessment therefore lies in scalability, whole-liver parenchymal sampling, and reduced operator dependency rather than in index-level diagnostic superiority, which would require further evaluation against functional or physiological reference standards.

This study has limitations. Training and testing were performed at a single center using consistent scanners and acquisition protocols. To address this limitation and confirm generalizability, multicenter external validation is warranted. Formal comparison of diagnostic performance between automated whole-liver and conventional ROI-based index calculation was not performed, as a functional or histological reference standard was not available for the randomly allocated radiological validation cohort. Reference segmentations were generated using a semi-automatic annotation workflow followed by slice-by-slice manual correction. This approach was chosen to ensure consistent annotations across a large multi-phase dataset, but it may not provide the same degree of independence as fully de novo manual contouring. Accordingly, DSCs and average surface distances should be interpreted in light of the semi-automatic reference standard. The muscle reference signal for LMR may be susceptible to pathological alterations, such as sarcopenia, myopathy, or fatty infiltration, which were not systematically assessed in this study. Liver indices requiring splenic normalization (HUI and LSR) were not applicable in patients with prior splenectomy, and these cases were excluded. The model was developed and validated across a broad spectrum of hepatic pathologies; however, similar to clinical radiological assessment, its performance may be limited in conditions with extensive replacement of normal liver parenchyma or marked anatomical distortion, such as diffuse infiltrative malignancy, polycystic liver disease, or post-surgical alterations including lobar resection. These scenarios were not systematically evaluated and warrant dedicated assessment in future studies.

In conclusion, phase-adaptive segmentation enables automated whole-liver quantification of SI-derived liver indices from gadoxetic acid-enhanced MRI without inter-phase co-registration, with excellent agreement with manual measurements and consistent differentiation between cirrhotic and non-cirrhotic patients based on HBP-derived liver indices. By providing reproducible indices across all DCE phases and reducing dependence on manual ROI measurements, this approach facilitates large-scale multi-phase clinical studies. Further validation against clinical reference standards, such as indocyanine green clearance and the ¹³C-methacetin breath test (LiMAx), including head-to-head comparison of automated *versus* manual approaches or composite scores such as the Functional Liver Imaging Score, is warranted to confirm these findings and support clinical translation.

## Supplementary information

**Additional file: Supplementary Table S1.** MRI acquisition parameters.** Supplementary Table S2.** Baseline characteristics of the development and test cohorts (n = 233). ** Supplementary Table S3.** Phase-Specific Segmentation Performance of TotalSegmentator MRI in the Test Cohort (500 Annotated Dynamic Contrast-Enhanced MRI Sequences). ** Supplementary Table S4.** Bland-Altman analysis of interreader and reader-model agreement for automated liver indices in the radiological validation cohort (n = 50). ** Supplementary Figure S1.** Challenging qualitative examples of model performance in edge cases. Representative cases illustrate model behaviour in anatomically and pathologically challenging scenarios, including axial hepatobiliary-phase T1-weighted fat-suppressed MRI images showing (a) extensive cystic liver disease with heterogeneous parenchymal appearance, resulting in incomplete segmentation of functional liver tissue, and (b) postoperative liver anatomy after left hemihepatectomy with additional segmental resection (segments VI/VII) and complex intrahepatic tumor burden, with misclassification of resection cavities as liver parenchyma and incomplete segmentation of the right posterolateral liver segments. These cases were selected to demonstrate robustness and expected limitations outside the main validated examples. ** Supplementary Figure S2.** TotalSegmentator MRI vs. Phase-Adaptive Model: Representative Segmentation Comparison Axial hepatobiliary-phase T1-weighted fat-suppressed MRI in two representative test-cohort patients. In each row, from left to right: the hepatobiliary-phase (HBP) image, the reference (ground-truth) segmentation, the TotalSegmentator MRI output, and the phase-adaptive model output. Liver parenchyma is shown in red and intrahepatic non-parenchymal structures in green. Both patients had undergone prior splenectomy. (a) The TotalSegmentator segmentation of liver parenchyma broadly includes intrahepatic non-parenchymal structures, such as vessels, and extends over the margins of metastases. In addition, portions of the caudate lobe and liver segments II and III adjacent to the liver surface are not segmented as part of the liver parenchyma. Additionally, TotalSegmentator MRI falsely labels residual splenic parenchyma (blue) despite prior splenectomy, whereas a single colorectal liver metastasis is correctly excluded from the parenchymal mask by the phase-adaptive model and no splenic tissue is detected. (b) Postoperative anatomy after left hemihepatectomy with residual tumor burden and reduced parenchymal volume; two ablation cavities and their adjacent parenchyma are correctly delineated only by the phase-adaptive model, in contrast to TotalSegmentator. ** Supplementary Figure S3.** Bland-Altman plots illustrating interreader agreement and agreement between readers and the model (automated pipeline) for signal intensity-derived liver indices in the radiological validation cohort (n = 50). Each row represents one index, and each column corresponds to a pairwise comparison: Reader 1 versus Reader 2 (left), Reader 1 versus model (middle), and Reader 2 versus model (right). In each plot, the x-axis shows the mean of both methods, and the y-axis shows their difference. The solid line indicates the mean bias, and the dashed lines indicate the 95% limits of agreement (bias±×±1.96 × SD). (a-c) Arterial enhancement fraction. (d-f) Relative liver enhancement (hepatobiliary phase [HBP], normalized). (g-i) Hepatocellular uptake index. (j-l) Liver-to-spleen ratio. (m-o) Liver-to-muscle ratio.

## Data Availability

The datasets generated and/or analyzed during the current study are not publicly available due to data protection regulations and patient privacy but are available from the corresponding author (FBO) on reasonable request.

## Abbreviations

AEF: Arterial enhancement fraction
AP: Arterial phase
DCE: Dynamic contrast-enhanced
DSC: Dice similarity coefficient
HBP: Hepatobiliary phase
HUI: Hepatocellular uptake index
ICC: Intraclass correlation coefficient
LMR: Liver-to-muscle ratio
LSR: Liver-to-spleen ratio
PC: Pre-contrast phase
PVP: Portal venous phase
RLE: Relative liver enhancement
ROI: Region of interest
SI: Signal intensity
VP: Venous phase
