# Supplementary material for: Phase-adaptive automated whole-liver quantification of signal intensity-derived liver indices from gadoxetic acid-enhanced MRI
Source: Eur Radiol Exp. 2026 Oct 12;10:155. doi: 10.1186/s41747-026-00813-z (PMC13658688; doi:10.1186/s41747-026-00813-z)
Supplement: Supplementary file 1 [file 41747_2026_813_MOESM1_ESM.pdf]

Phase-Adaptive Automated Whole-Liver Quantification of Signal Intensity-Derived Liver Indices from Gadoxetic Acid-Enhanced MRI

ELECTRONIC SUPPLEMENTARY MATERIAL

Supplementary Table S1. MRI acquisition parameters

**Surface coil**	**Multielement 16-channel coil (Sense Torso XL)**
**Type of scanner**	1.5 T Ingenia or 1.5 T Ambition (Philips Healthcare)
	Dynamic series
**Pulse sequence type**	T1-weighted mDixon-Dynamic
**TR/TE [ms]**	5.3/1.7
**Flip angle**	15°
**Orientation**	transverse
**Matrix scan**	288 (reconstruction: 512)
**Field of view (FOV)**	330 mm
**Slice thickness**	5 mm
**Acquired Voxel**	1.35 x 1.71 x 5.00 mm
**Reconstructed Voxel**	0.76 x 0.76 x 2.5 mm
**Breath compensation**	Breath-hold
**Compressed Sense factor**	2
**Dynamic phases**	PC, AP, PVP, VP, HBP
**Duration**	~ 1 min (4x ~ 18 seconds: pre-contrast, AP, PVP and VP); ~ 18 seconds (HBP)

*Dynamic contrast-enhanced MRI was performed after gadoxetic acid administration during the arterial (AP, ~ 10 seconds), portal venous (PVP, ~30-35 seconds), venous (VP, ~75 seconds), and hepatobiliary (HBP, ~20 minutes) phases. Abbreviations: PC, pre-contrast phase; AP, arterial phase; HBP, hepatobiliary phase; PVP, portal venous phase; VP, venous phase.

Supplementary Table S2. Baseline characteristics of the development and test cohorts (n = 233)

Characteristic	Overall (n = 233)	Development cohort (n = 133)	Test cohort (n = 100)	*p*-value
Age (years), mean ± SD (range)	60.3 ± 13.2 (19-87)	60.7 ± 13.5 (19-87)	59.7 ± 12.8 (19-85)	0.55
Male sex	133 (57.1%)	78 (58.6%)	55 (55.0%)	0.67
Cirrhosis	26 (11.2%)	18 (13.5%)	8 (8.0%)	0.26
Previous liver surgery	172 (73.8%)	93 (69.9%)	79 (79.0%)	0.16
Hemihepatectomy	26 (11.2%)	13 (9.8%)	13 (13.0%)	0.57
Benign hepatic lesion	108 (46.4%)	67 (50.4%)	41 (41.0%)	0.20
Malignant hepatic lesion	121 (51.9%)	62 (46.6%)	59 (59.0%)	0.08
Hepatic lesion burden				0.10
0	26 (11.2%)	15 (11.3%)	11 (11.0%)	
1	39 (16.7%)	21 (15.8%)	18 (18.0%)	
2	68 (29.2%)	45 (33.8%)	23 (23.0%)	
3	17 (7.3%)	5 (3.8%)	12 (12.0%)	
Multiple	83 (35.6%)	47 (35.3%)	36 (36.0%)	
Splenectomy	6 (2.6%)	2 (1.5%)	4 (4.0%)	0.41

SD, standard deviation.

Data are numbers of patients, with percentages in parentheses, unless otherwise indicated. The development cohort comprised 133 patients, yielding 403 annotated dynamic contrast-enhanced (DCE) MRI sequences, and the test cohort comprised 100 patients and 500 annotated DCE-MRI sequences. *P*-values refer to comparisons between the development and test cohorts. For categorical variables with more than two categories, *p*-values represent global comparisons across all categories.

Supplementary Table S3. Phase-Specific Segmentation Performance of TotalSegmentator MRI in the Test Cohort (500 Annotated Dynamic Contrast-Enhanced MRI Sequences)

	**Dice similarity coefficient**	**Average surface distance [mm]**
**Pre-contrast phase**		
Liver parenchyma	0.951 [0.944-0.957]	2.134 [1.866-2.309]
Spleen*	0.938 [0.926-0.950]	0.729 [0.604-0.998]
**Arterial phase**		
Liver parenchyma	0.949 [0.938-0.955]	2.363 [1.956-2.830]
Spleen*	0.937 [0.908-0.954]	0.684 [0.513-1.202]
**Portal venous phase**		
Liver parenchyma	0.943 [0.934-0.951]	2.633 [2.183-3.043]
Spleen*	0.945 [0.923-0.957]	0.644 [0.473-1.004]
**Venous phase**		
Liver parenchyma	0.950 [0.936-0.956]	1.751 [1.378-2.388]
Spleen*	0.949 [0.935-0.960]	0.582 [0.418-0.778]
**Hepatobiliary phase**		
Liver parenchyma	0.951 [0.942-0.956]	2.462 [2.113-2.760]
Spleen*	0.942 [0.910-0.942]	0.899 [0.707-1.172]

Data are median [interquartile range].

The test cohort comprised 100 patients with complete phase coverage and 500 annotated dynamic contrast-enhanced MRI sequences. Dice similarity coefficient values range from 0 to 1, with higher values indicating better overlap. Average surface distance is reported in millimeters, with lower values indicating better boundary agreement.

The composite non-parenchymal class (intrahepatic vessels, bile ducts, focal liver lesions, and non-functional post-treatment changes including resection cavities and ablation zones) is not among the TotalSegmentator MRI labels; segmentation performance for this class could therefore not be evaluated for TotalSegmentator MRI and is not reported in this table.

*Spleen segmentation metrics were calculated in 96 patients (480 annotated dynamic contrast-enhanced MRI sequences) because four patients had prior splenectomy.

Supplementary Table S4. Bland-Altman analysis of interreader and reader-model agreement for automated liver indices in the radiological validation cohort (n = 50)

	Bias (mean of differences)			Standard deviation of differences			95% limits of agreement [lower, upper]		
**Index**	**Reader 1 − Reader 2**	**Model − Reader 1**	**Model− Reader 2**	**Reader 1 − Reader 2**	**Model − Reader 1**	**Model − Reader 2**	**Reader 1 − Reader 2**	**Model − Reader 1**	**Model − Reader 2**
Arterial enhancement fraction	−0.5	1.0	0.7	5.3	6.6	7.0	[−10.7, 9.9]	[−11.9, 13.9]	[−12.9, 14.3]
Relative liver enhancement (HBP, normalized)	0.004	0.005	0.009	0.07	0.06	0.08	[−0.135, 0.142]	[−0.111, 0.122]	[−0.146, 0.164]
Hepatocellular uptake index	46	−104	−59	100	133	141	[−149, 241]	[−366, 157]	[−335, 218]
Liver-to-spleen ratio	0.035	−0.078	−0.044	0.07	0.09	0.10	[−0.101, 0.171]	[−0.254, 0.099]	[−0.232, 0.144]
Liver-to-muscle ratio	−0.004	−0.107	−0.110	0.09	0.11	0.13	[−0.183, 0.174]	[−0.330, 0.116]	[−0.364, 0.145]

HBP, hepatobiliary phase.
Bias is the mean of pairwise signed differences across patients. Standard deviation (SD) reflects dispersion. Limits of agreement are calculated as bias ± 1.96 × SD. The radiological validation cohort comprised 50 patients. MRI-derived indices were computed per patient from phase-specific signal intensity values obtained from manual regions of interest (Reader 1 and Reader 2) and the parenchyma-only liver mask generated by the model.

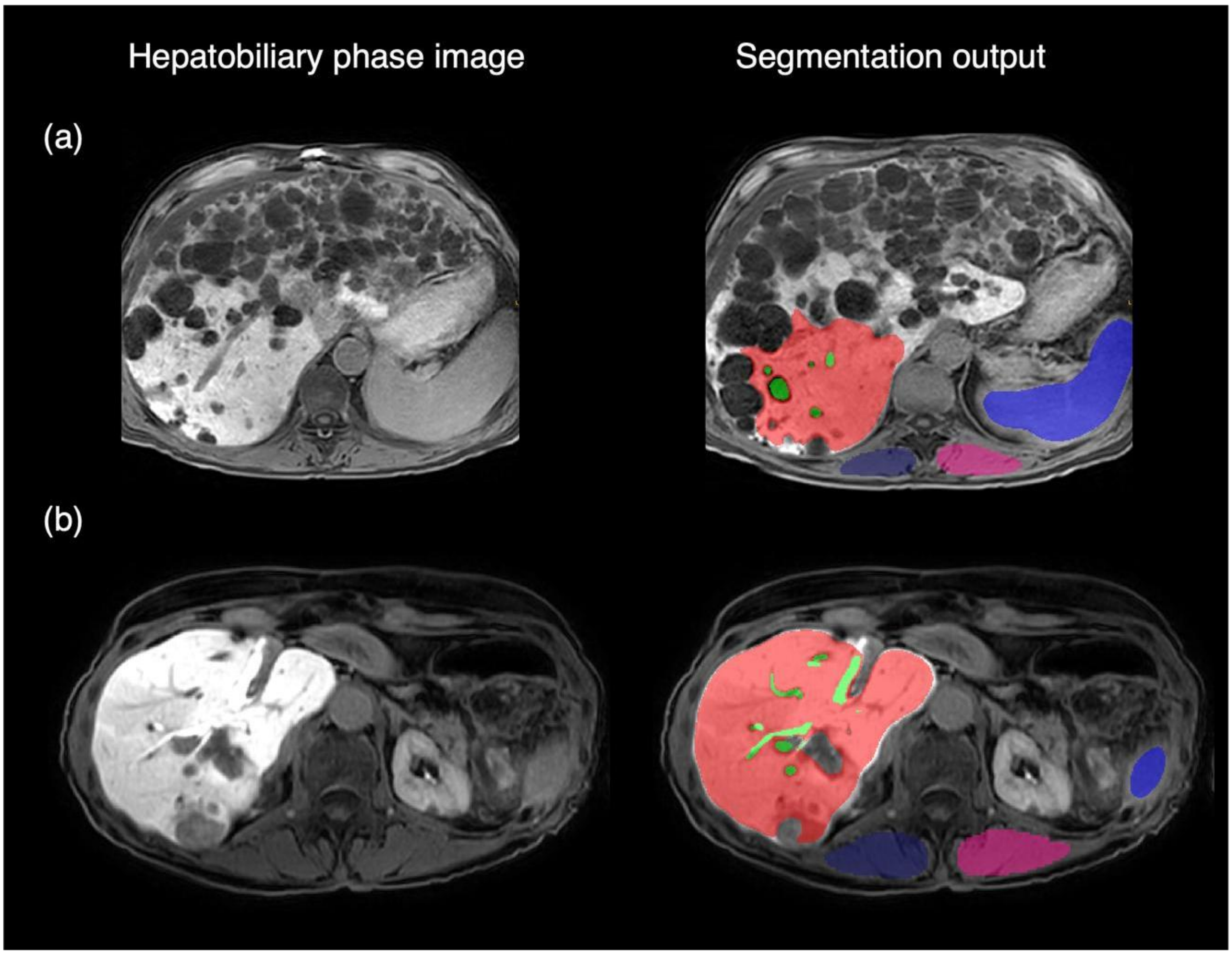

Supplementary Figure S1. Challenging qualitative examples of model performance in edge cases. Representative cases illustrate model behaviour in anatomically and pathologically challenging scenarios, including axial hepatobiliary-phase T1-weighted fat-suppressed MRI images showing (a) extensive cystic liver disease with heterogeneous parenchymal appearance, resulting in incomplete segmentation of functional liver tissue, and (b) postoperative liver anatomy after left hemihepatectomy with additional segmental resection (segments VI/VII) and complex intrahepatic tumor burden, with misclassification of resection cavities as liver parenchyma and incomplete segmentation of the right posterolateral liver segments. These cases were selected to demonstrate robustness and expected limitations outside the main validated examples.

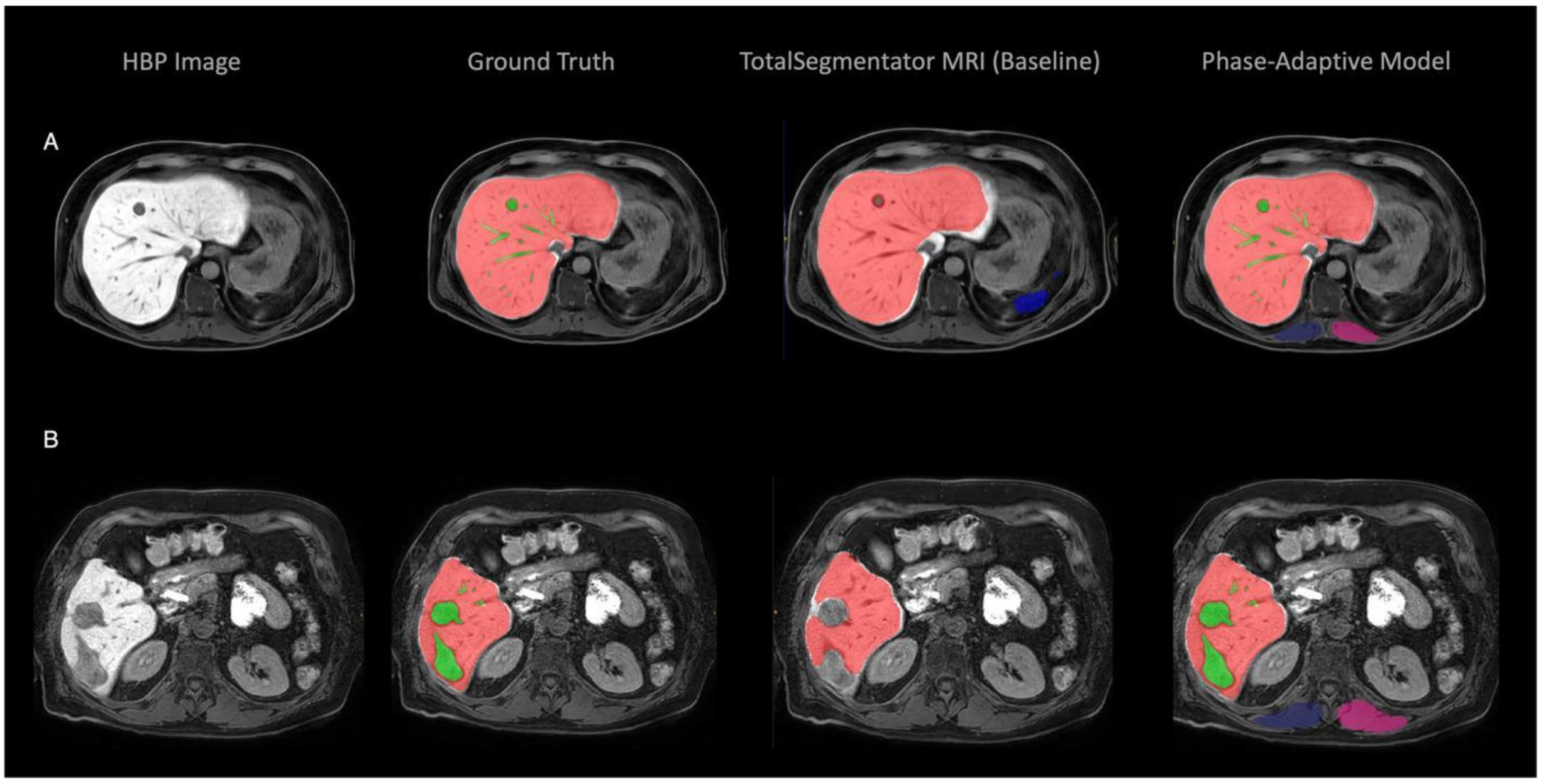

Supplementary Figure S2. TotalSegmentator MRI vs. Phase-Adaptive Model: Representative Segmentation Comparison

Axial hepatobiliary-phase T1-weighted fat-suppressed MRI in two representative test-cohort patients. In each row, from left to right: the hepatobiliary-phase (HBP) image, the reference (ground-truth) segmentation, the TotalSegmentator MRI output, and the phase-adaptive model output. Liver parenchyma is shown in red and intrahepatic non-parenchymal structures in green. Both patients had undergone prior splenectomy. (a) The TotalSegmentator segmentation of liver parenchyma broadly includes intrahepatic non-parenchymal structures, such as vessels, and extends over the margins of metastases. In addition, portions of the caudate lobe and liver segments II and III adjacent to the liver surface are not segmented as part of the liver parenchyma. Additionally, TotalSegmentator MRI falsely labels residual splenic parenchyma (blue) despite prior splenectomy, whereas a single colorectal liver metastasis is correctly excluded from the parenchymal mask by the phase-adaptive model and no splenic tissue is detected. (b) Postoperative anatomy after left hemihepatectomy with residual tumor burden and reduced parenchymal volume; two ablation cavities and their adjacent parenchyma are correctly delineated only by the phase-adaptive model, in contrast to TotalSegmentator.

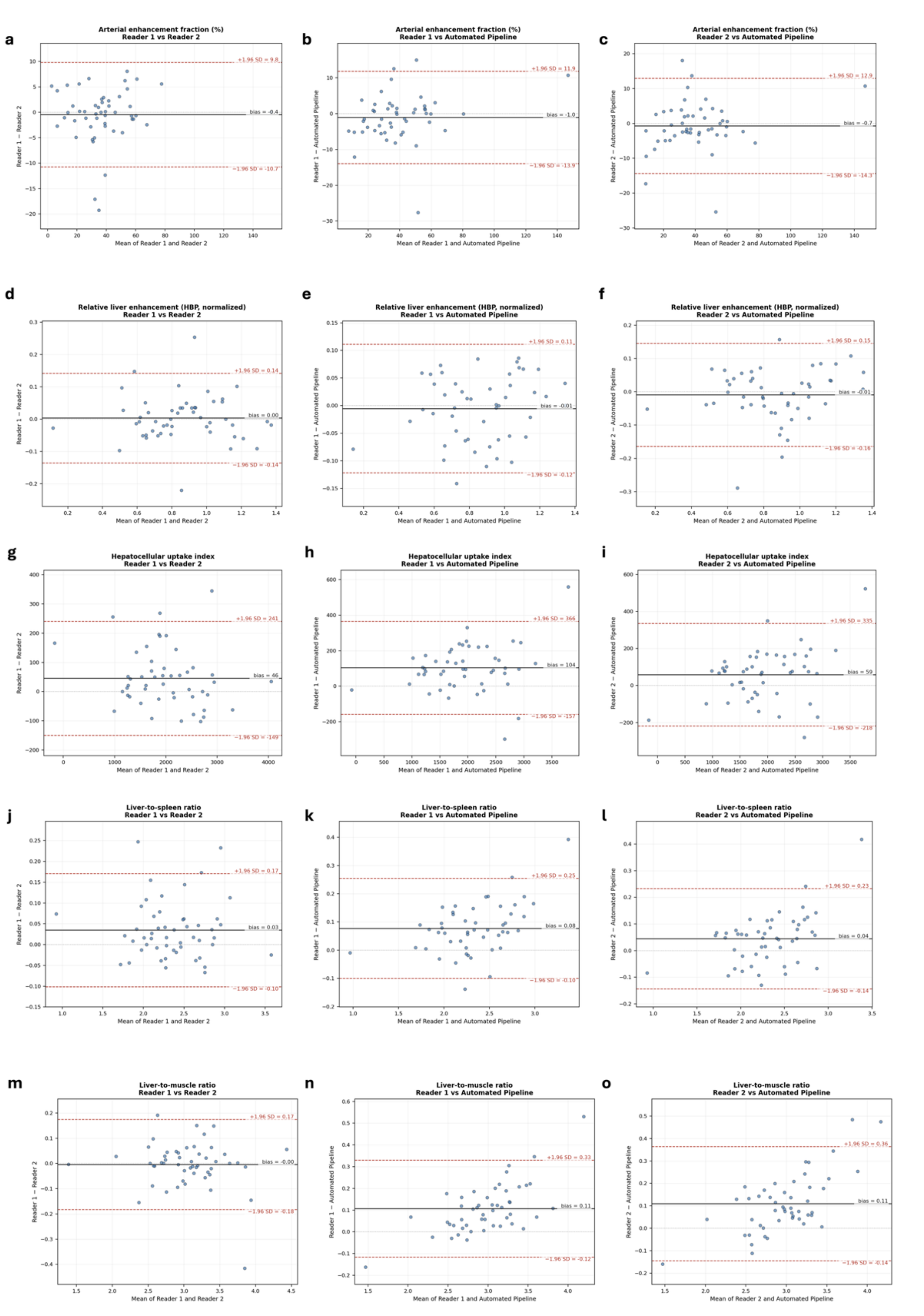

Supplementary Figure S3. Bland-Altman plots illustrating interreader agreement and agreement between readers and the model (automated pipeline) for signal intensity-derived liver indices in the radiological validation cohort (n = 50). Each row represents one index, and each column corresponds to a pairwise comparison: Reader 1 versus Reader 2 (left), Reader 1 versus model (middle), and Reader 2 versus model (right). In each plot, the x-axis shows the mean of both methods, and the y-axis shows their difference. The solid line indicates the mean bias, and the dashed lines indicate the 95% limits of agreement (bias ± 1.96 × SD). (a-c) Arterial enhancement fraction. (d-f) Relative liver enhancement (hepatobiliary phase [HBP], normalized). (g-i) Hepatocellular uptake index. (j-l) Liver-to-spleen ratio. (m-o) Liver-to-muscle ratio.
